# LncRNA WDFY3-AS2 promotes cisplatin resistance and the cancer stem cell in ovarian cancer by regulating hsa-miR-139-5p/SDC4 axis

**DOI:** 10.1186/s12935-021-01993-x

**Published:** 2021-05-29

**Authors:** Yue Wu, Ting Wang, Lin Xia, Mei Zhang

**Affiliations:** 1 Department of Integrated Chinese and Western Medicine Oncology, The First Affiliated Hospital of Anhui Medical University, No. 218, Jixi Road, Hefei, 230022 Anhui China; 2 The Traditional and Western Medicine (TCM)-Integrated Cancer Center of Anhui Medical University, 81 Meishan Road, Hefei, 230032 China; 3 Graduate School of Anhui, University of Traditional Chinese Medicine, Hefei, 230012 Anhui China

**Keywords:** lncRNA WDFY3-AS2, Hsa-miR-139-5p, SDC4, Cisplatin resistance, Cancer stem cells, Ovarian cancer

## Abstract

**Background:**

Ovarian cancer (OC) is a high-mortality gynecological cancer that is typically treated with cisplatin, although such treatment often results in chemoresistance. Ovarian cancer resistance is usually related to cell stemness. Herein, we explored the function of lncRNA WDFY3-AS2 in OC cell resistance to cisplatin (DDP).

**Methods:**

Cisplatin resistant OC A2780 cell lines (A2780-DDP) were established by long-term exposure to cisplatin. CCK-8 assay were performed to evaluate the viability of A2780, and A2780-DDP cells. Quantitative RT-PCR was used to examine the expression of lncRNA WDFY3-AS2, miR-139-5p, and SDC4 in A2780-DDP cell lines. After treatment with cisplatin, cell apoptosis and CD44^+^CD166^+^-positive cells were measured by flow cytometry. The transwell assays were employed to measure the effect of WDFY3-AS2 on cell migration, and invasion. In addition, tumorsphere formation assay was used to enrich OC cancer stem cells (CSCs) from A2780-DDP cells. The expression of CSC markers (SOX2, OCT4, and Nanog) was detected by western blotting. The regulatory mechanism was confirmed by RNA pull down, and luciferase reporter assays. Furthermore, xenograft tumor in nude mice was used to assess the impact of WDFY3-AS2 on cisplatin resistance in OC in vivo.

**Results:**

WDFY3-AS2 was highly expressed in OC A2780-DDP cells, and silencing WDFY3-AS2 significantly inhibited proliferation, migration and invasion but increased apoptosis in OC A2780-DDP cells. Additionally, WDFY3-AS2 significantly promoted the A2780-DDP cells tumorspheres. WDFY3-AS2 was predicted to impact OC by sponging miR-139-5p and regulating SDC4. The xenografts inoculated with A2780-DDP cells additionally confirmed that tumor growth in vivo was reduced by si-WDFY3-AS2 transfection. MiR-139-5p inhibitor or SDC4 overexpression could restore the suppressive influence of silenced WDFY3-AS2 on tumor growth.

**Conclusions:**

Together, WDFY3-AS2 may lead to change of cisplatin resistance by the expression of miR-139-5p/SDC4 in the OC A2870-DDP cells both in vitro and in vivo. Our finding may provide a drug target for the drug resistance of OC.

**Supplementary Information:**

The online version contains supplementary material available at 10.1186/s12935-021-01993-x.

## Background

Ovarian cancer (OC) is among the most prevalent and deadliest gynecological malignancies globally [1], and can arise in the epithelial ovarian cancer (EOC) and ovarian cancer of germ cell origin subtypes [2, 3], with EOC being more common and with the epithelium being the main site of tumor growth [4]. Combinations of surgery, chemotherapy and radiotherapy are the main means of clinically treating OC [5]. The prognosis of OC is very poor on account of late diagnosis and limited effective treatment; it is mainly because of chemotherapy resistance post-surgery [6]. The mechanisms governing OC therapeutic resistance thus warrant further study.

Cancer stem cells (CSCs) are important drivers of therapeutic resistance in many tumor types [7], and represent a population of tumor cells that exhibit stem-like properties including the ability to differentiate and undergo self-renewal, thus allowing them to support sustained tumor growth and heterogeneity [8, 9]. Owing to their properties, CSCs are thought to be important drivers of tumor metastasis and other malignant processes [10, 11], and developing approaches to eliminating these cells is therefore vital to decrease the odds of OC therapeutic resistance or relapse [12].

The molecular networks are found to provide targeted approaches to treating OC development [13]. Non-coding RNAs including microRNAs (miRNAs) and long noncoding RNAs (lncRNAs) have been characterized in recent years as key regulators of gene expression and may be viable targets of therapeutic intervention in OC [14]. LncRNAs are > 200 nt long and do not encode proteins [15], yet can post-transcriptionally regulate diverse biological processes in many forms of cancer [16–18]. Increasing evidences have shown lncRNAs to control invasion, metastasis, differentiation, apoptosis, cell cycle progression, and tumor development [19–22]. An increasing number of lncRNAs, including lncRNA MEG3, lncRNA UCA1, and lncRNA LINC00511, are related to OC onset and progression [23–25]. The previous report revealed that WDFY3-AS2 suppressed ovarian cancer progression by sponging miR-18a [26], WDFY3-AS2 may suppress the proliferation and invasion in oesophageal squamous cell carcinoma by regulating miR-2355-5p/SOCS2 axis [27], and WDFY3-AS2 also was reported to promote lung adenocarcinoma progression via targeting miR-491-5p/ZNF703 axis [28]. These findings highlighted the role of WDFY3-AS2 in tumour development and progression, however, too little is known regarding the role of WDFY3-AS2 in cisplatin resistance of OC. Also, the understanding of their mechanisms in the tumor stem cell is inadequate. These topics thus warrant further detailed study.

Syndecan (SDC) proteoglycans, which include SDC1-4, are linked to key proliferative, migratory, and differentiation processes in cells [29]. Many cells express SDC4, which has been reported to influence ovarian carcinoma cells [30].

In light of these prior reports, this study sought to explore the modulatory effects of lncRNA WDFY3-AS2 in OC drug resistance. Since lncRNAs can usually be used as miRNA molecular sponges to regulate miRNA target gene expression, in this experiment, the ceRNA mechanism was used as the main mechanism for mediating lncRNA WDFY3-AS2 to regulate drug resistance in OC. In our study, we also demonstrate that lncRNA WDFY3-AS2 function as a sponge of hsa-miR-139-5p which in turn regulates the expression of SDC4 on tumorigenic function of CSCs and will provide crucial information for its application for the treatment of OC.

## Methods

### Sample collection

OC tumor and paracancerous tissues were collected from 30 patients at The First Affiliated Hospital of Anhui Medical University from March 2019–October 2019. All patients had provided written informed consent, and none had undergone preoperative radiotherapy or chemotherapy. The Ethics Committee of The First Affiliated Hospital of Anhui Medical University approved this study (PJ2019-03-19). After collection, samples were stored at − 80 °C, for further experiments.

### Cells culture and treatment

Human OC cell lines A2780, A2780/DDP (cisplatin-resistant A2780 cells) were from American Type Culture Collection (ATCC, USA). Cells were grown routinely in RPMI-1640 (Invitrogen, CA, USA) containing 10% FBS (Gibco, CA, USA) and 100 U/mL penicillin/streptomycin in a 37 °C humidified 5% CO_2_ incubator.

### Cell transfection and grouping

The pcDNA3.1/WDFY3-AS2 (WDFY3-AS2), pcDNA3.1/NC (vector), si-WDFY3-AS2 or si-NC was prepared by GenePharma (Shanghai, China), as were the hsa-miR-139-5p mimic, mimic control (miR-NC), hsa-miR-139-5p inhibitor, and inhibitor control (inhibitor-NC) constructs. For gene RNAi, pcDNA3.1/SDC4 (SDC4) and pcDNA3.1/NC (pcDNA-NC) were obtained from GenePharma. For transfection reactions, cells were added to 6-well plates (1 × 10^5^/well) and Lipofectamine 3000 (Thermo Fisher Scientific, MA, USA) was used based on provided directions. At 48 h post-transfection, cells were collected for downstream analysis.

### qRT-PCR

Total RNA samples were obtained using RNA extraction kit (Biomed, Beijing, China). Sample concentrations were measured using Nanovue spectrophotometer (GE, Buckinghamshire, UK). RNA was used with a First-Stand cDNA Synthesis Super Mix (TransGen Biotech. Beijing, China) to prepare cDNA. These were then analyzed by qRT-PCR (Applied Biosystems, Carlsbad, USA) using TransStart Green qPCR Super Mix (TransGen Biotech, Beijing, China). GAPDH was used as an internal control. Primers for lncRNA WDFY3-AS2, hsa-miR-139-5p, SDC4, GAPDH used in this study were as follows: WDFY3-AS2 (F: 5′-GAAACGCAAAGGCTACTAGAC-3′, R: 5′-AGTTTCTTTCCATCTGGTCCT-3′), the mature hsa-miR-139-5p (F: 5′-TCTACAGTGCACGTGTCTCCAGT-3′, R: 5′-TGGAGACACGTGCACTGTAGATT-3′), U6 (F: 5′-CTCGCTTCGGCAGCACA-3′, R: 5′-AACGCTTCACGAATTTGCGT-3′), SDC4 (F: 5′-CGATGAGGATGTAGTGGGGC-3′, R: 5′-GACAACTTCAGGGCCGATCA-3′), GAPDH (F: 5′-GAAGGTGAAGGTCGGAGTC-3′, R: 5′-GAAGATGGTGATGGGATTTC-3′). Thermocycler settings were: initial denaturation at 95 °C for 10 min; 40 cycles of 95 °C for 15 s; annealing at 67 °C for 30 s; and a final extension at 72 °C for 30 s. Samples were analyzed in triplicate, with the 2^−ΔΔCt^ method being used to quantify relative gene expression (Additional file 1).

### CCK-8 assay

The cells were treated with cisplatin at variant concentrations (0, 5, 10, 15, 20, 25, 30 μΜ) for 72 h. In addition, cells treated with 20 μΜ cisplatin were planted into 96-well plates. A CCK-8 assay performed as previously described [31] was used to measure viability. In brief, After 24, 48 and 72 h, 10 μL CCK-8 reagent was applied to stain the cells. The optical density (OD) was measured at 450 nm with an enzyme-linked immunometric meter (mode 680, Bio-Rad, Hercules, USA).

### Transwell assays for migration and invasion

Transwell assays were performed by using transwell chambers (Corning, USA). After 48 h transfection, OC A2780-DDP treated with 20 μΜ cisplatin, cells migration and invasion were detected as described in the past [26].

### Flow cytometry for cell apoptosis

An Annexin V-FITC kit (Biosea Biotechnology Co., Beijing, China) was used as previously described [31] to measure cell apoptosis.

### Tumorsphere formation assay

The transfected cells with 20 μM cisplatin 48 h treatment were cultured in serum-free DMEM/F12 containing 20 ng/mL EGF, 20 ng/mL bFGF, and B27. Following plating into 24-well ultra-low attachment plates (5000 cells/well), growth media was changed every 2 days and light microscopy (Nikon, Japan) was used to monitor tumorspheres.

### Cell surface marker analysis (CD44, CD166)

The CD44 and CD166 antibody were obtained from Beijing biosynthesis biotechnology CO., LTD. The transfected cells after treatment with 20 μM cisplatin were cultured in serum-free medium (SFM). After a 7-day period, the number of CD44^+^ and CD166^+^ positive cells was assessed via flow cytometry assay. Detection of CD44^+^ and CD166^+^ positive cells was carried out according to provided directions.

### Western blotting

RIPA buffer (Sigma, USA) was used to isolate protein from rat lung tissue samples, and there proteins were then separated by 10% SDS-PAGE, transferred to PVDF membranes (Millipore, MA, USA), and these were blocked for 2 h with 5% non-fat milk, followed by probing overnight with anti-GAPDH (ab8245, Abcam, UK), anti-SDC4 (ab74139, Abcam), anti-SOX4 (ab70598, Abcam), anti-OCT4 (ab200834, Abcam), and anti-Nanog (ab109250, Abcam), all diluted 1:1000. Following three washes, blots were probed with HRP-anti-rabbit IgG (1:2000, ab6721, Abcam) for 2 h, after which bands were detected using an enhanced chemiluminescence system (ECL, ThermoFisher, USA), with the Image Lab™ Software (Bio-Rad, USA) being used to analyze the data.

### Dual-luciferase reporter assay

Binding interactions between the lncRNA WDFY3-AS2 and hsa-miR-139-5p, between the SDC4 and hsa-miR-139-5p were predicted using the Starbase v2.0 (http://starbase.sysu.edu.cn/) to identify sites of predicted sequence complementarity. The pmirGLO vectors containing WT or mutated WDFY3-AS2 (WDFY3-AS2-WT, WDFY3-AS2-MUT) or SDC4 (SDC4-WT, SDC4-MUT) binding site in miR-139-5p were constructed. Cells were then transfected with these plasmids via the use of Lipofectamine 3000, and a dual-luciferase kit (Promega, USA) was used to quantify luciferase activity.

### RNA pull-down assay

Biotinylated WDFY3-AS2 probe, miR-139-5p probe, and corresponding controls were obtained from GenePharma (Shanghai, China). Cellular lysates were combined with M-280 streptavidin magnetic beads (Invitrogen) as discussed in prior studies [20], and qRT-PCR was used to detect the miR-139-5p or SDC4 expression.

### In vivo tumorigenesis assay

All BALB/C female nude mice (6 weeks old) were purchased from the Changzhou card Vince laboratory animal Co., Ltd (SCXK(Su) 2011-0003, Changzhou, China). Centre (ARASC), Health Campus, Universiti Sains Malaysia (USM). Mice were housed in specific pathogen-free facility with a 12 h light cycle and food and water ad libitum in Anhui Medical University. Animals studies were consistent with the NIH Guide for the Care and Use of Laboratory Animals and received approval from the Animal Care and Use Committee of the First Affiliated Hospital of Anhui Medical University. The A2780-DDP cells (3 × 10^6^, 200 μL) transfected with si-WDFY3-AS2, si-WDFY3-AS2 + inhibitor-NC, or si-WDFY3-AS2 + miR-139 inhibitor, si-WDFY3-AS2 + pcDNA-NC, si-WDFY3-AS2 + SDC4, and control were subcutaneously implanted in the right flank of nude mice (9 mice per group). Subsequently, cisplatin, at a dose of 2.5 mg/kg/2 days, was administrated into the abdominal cavity of the nude mice. Every 7 days, the growth of these tumors was assessed, with volume being quantified as follows: volume = (length × width^2^)/2 (mm^3^). Mice were anesthetized by exposure to 1–3% isoflurane 4 weeks following tumor implantation, at which time these tumors were collected, weighed, and imaged.

### Statistical analysis

GraphPad Prism 7.0 (GraphPad, CA, USA) and SPSS 22.0 (SPSS Inc. IL, USA) were used for analyzing data, which are given as means ± SD (standard deviation) and were compared via one-way ANOVAs with Tukey’s post hoc tests. All experiments were carried out in triplicate, and P < 0.05 was the significance threshold.

## Results

### The levels of lncRNA WDFY3-AS2 in OC tissues and cells with different levels of cisplatin sensitivity

**Fig. 1:**
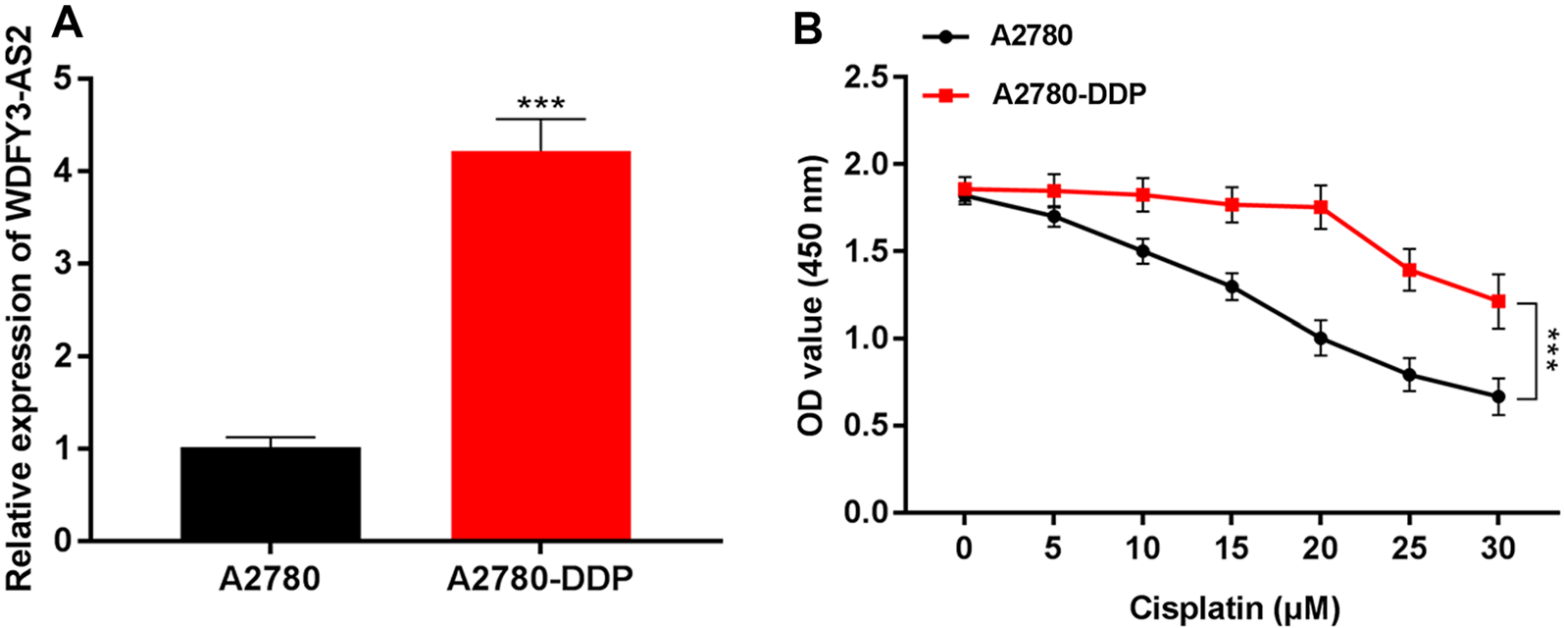
LncRNA WDFY3-AS2 expression was increased in cisplatin-resistant OC. **A** qRT-PCR expression analysis of LncRNA WDFY3-AS2 in OC cell (A2780-DDP cells and A2780 cells). **B** Detection of cell ability of OC cells with (0, 5, 10, 15, 20, 25, 30 μM) cisplatin treatment by CCK-8 assay. ***P < 0.001

qRT-PCR revealed that WDFY3-AS2 expression patterns in OC cell line were as follows: the expression of WDFY3-AS2 in A2780-DDP cells was significantly higher than that in A2780 cells (Fig. 1A, P < 0.001), suggesting higher WDFY3-AS2 levels in cisplatin-resistant OC cells compared to cisplatin-sensitive OC cells. Furthermore, to determine whether lncRNA WDFY3-AS2 was involved in cisplatin resistance in OC, firstly, CCK-8 assay showed that in cisplatin-resistant cells (A2780-DDP) and A2780 cells, after cisplatin treatment with an increasing concentration (0, 5, 10, 15, 20, 25, 30 μM), the viability of cisplatin-sensitive cells was reduced with the increase of cisplatin concentration. Cisplatin-resistant cells had no significant changes in cell viability under the treatment of 0–20 μM cisplatin, whereas the viability of cisplatin-resistant cells was decreased under the treatment of 20–30 μM (P < 0.001, Fig. 1B). A 20 μM cisplatin dose was thus used in subsequent analyses of A2780 cells.

### The effect of lncRNA WDFY3-AS2 on cisplatin resistance and CSCs of OC

**Fig. 2:**
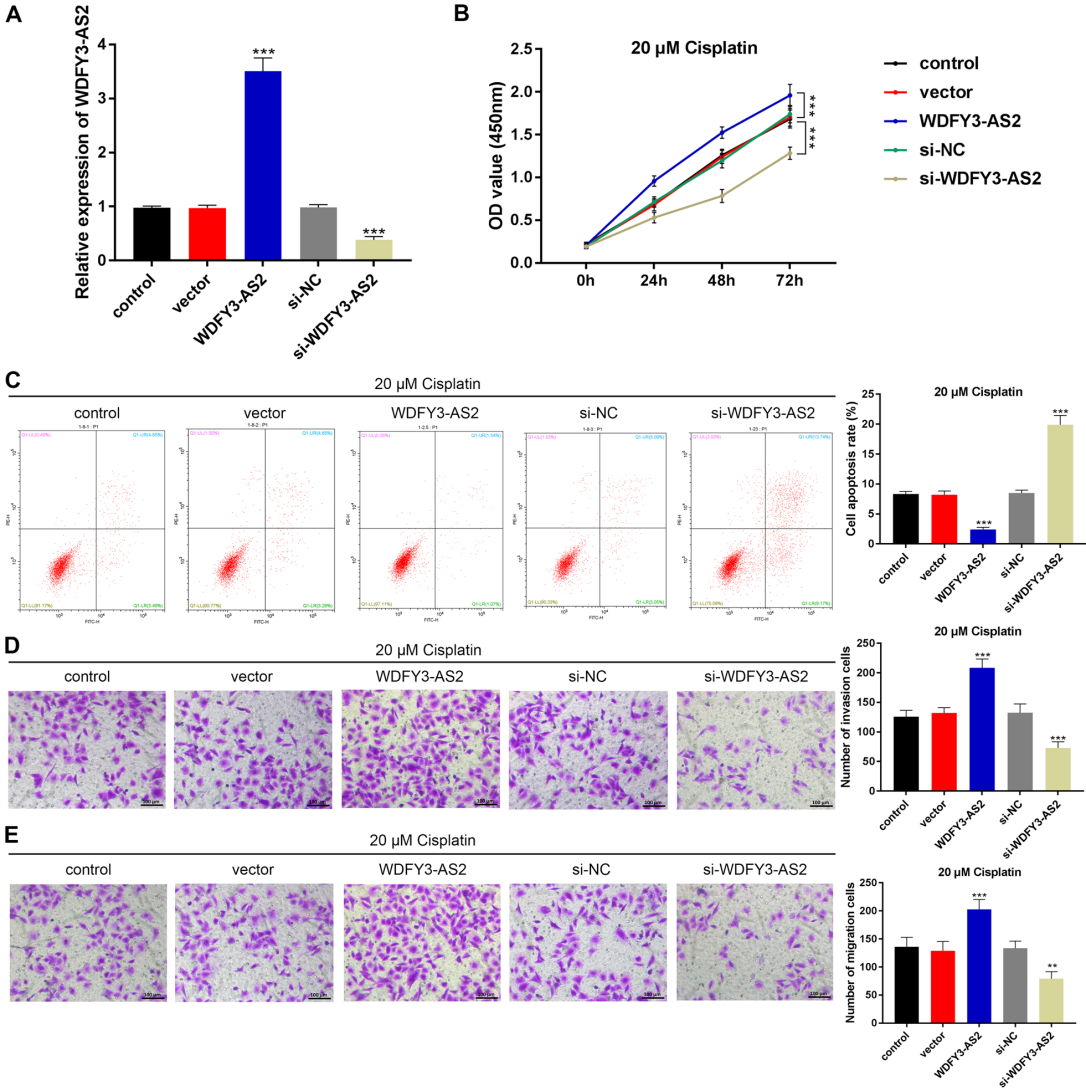
LncRNA WDFY3-AS2 promoted the cisplatin-resistant in OC cells. **A** The transfection efficiency of lncRNA WDFY3-AS2 in OC A2780-DDP cells. **B** CCK8 results of the cell viablity of transfected A2780-DDP cells with cisplatin (20 μM). **C** Flow cytometric detection of apoptosis of A2780-DDP cells with cisplatin (20 μM). **D**, **E** Transwell assay indicated that the invasive and migration activity in A2780-DDP cells with (20 μM) cisplatin treatment (100 μm). ***P < 0.001 vs. control

We sought to confirm the biological role of WDFY3-AS2 in the progression of OC. Respective plasmids pcDNA3.1/lncRNA WDFY3-AS2 (WDFY3-AS2), pcDNA-NC, lncRNA WDFY3-AS2 siRNA (si-WDFY3-AS2) and si-NC were transfected into A2780-DDP cells, with subsequent qRT-PCR analyses (Fig. 2A) showing that WDFY3-AS2 expression rose in WDFY3-AS2 transfected cells, relative to the pcDNA-NC transfected cells (P < 0.001). Whereas si-WDFY3-AS2 markedly inhibited WDFY3-AS2 expression in A2780 cells (P < 0.001). CCK-8 assay illustrated that in cisplatin-resistant A2780-DDP cells, after the cisplatin treatment (20 μM), the cell viability was notably enhanced after WDFY3-AS2 transfection (P < 0.001). However, the cell viability was significantly decreased after transfection of si-WDFY3-AS2 (P < 0.001, Fig. 2B). In A2780-DDP cells with cisplatin treatment (20 μM), cell apoptosis displayed an obvious decrease in the presence of WDFY3-AS2, following the transfection of si-WDFY3-AS2, cell apoptotic rate was obviously increased, as shown by flow cytometry (all P < 0.001, Fig. 2C).

To additionally assess the function of WDFY3-AS2 in cisplatin-resistant OC cell migration and invasion, Transwell assays were conducted. A2780-DDP cells after cisplatin treatment (20 μM). The data showed that WDFY3-AS2 significantly enhanced both invasion and migration (P < 0.001); in contrast, si-WDFY3-AS2 overexpression markedly suppressed cell invasion and migration (P < 0.001, Fig. 2D, E). Taken together, these data indicate that WDFY3-AS2 could induce resistance to cisplatin in OC A2780-DDP cells.

### The impact of WDFY3-AS2 on CSCs of OC

**Fig. 3:**
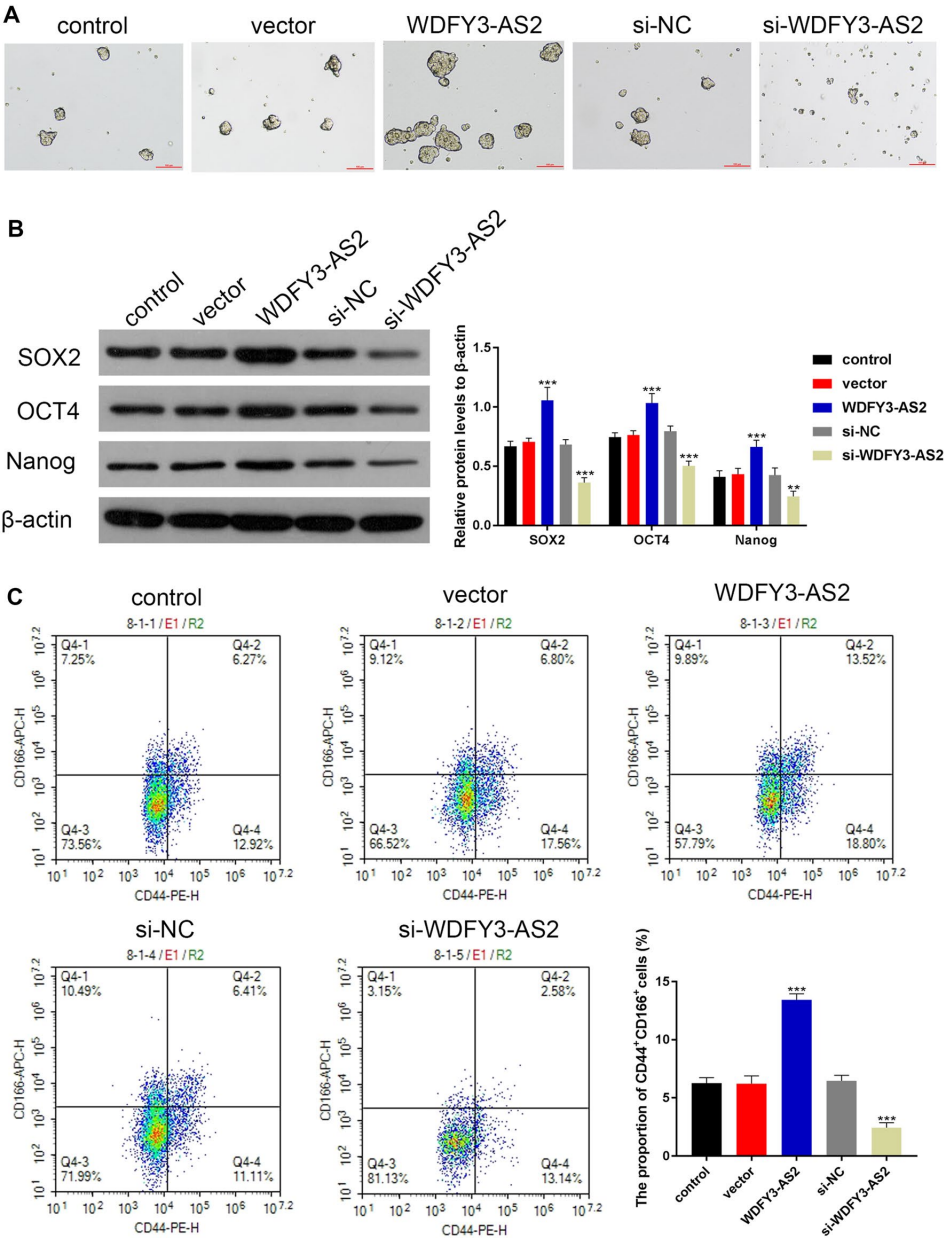
LncRNA WDFY3-AS2 induced the traits of CSCs. **A** Representative A2780-DDP sphere-forming cells were imaged (100 μm). A2780-DDP cells were treated for 7 days with 20 μM cisplatin. **B** Western blotting was used to detect the CSC markers protein expression including the SOX2, OCT4 and Nanog in A2780-DDP sphere-forming cells. **C** Detection of CD44^+^, CD166^+^-positive cells in A2780-DDP sphere-forming cells via flow cytometry. **P < 0.01 vs. control, ***P < 0.001 vs. control

To examine whether WDFY3-AS2 was able to suppress OC CSCs, we treated the transfected sphere-forming cells with 20 μM concentrations of cisplatin. Seven days post-treatment, WDFY3-AS2 overexpression promoted the formation of A2780-DDP tumorspheres; in contrast, si-WDFY3-AS2 significantly inhibited the formation tumorspheres (Fig. 3A). In addition, we examined the expression of stem cell marker proteins SOX2, OCT4 and Nanog by Western blot analysis. After 7 days of SFM culture, A2780-DDP sphere-forming cells with the WDFY3-AS2 transfection expressed markedly higher levels of CSC markers including the SOX2, OCT4 and Nanog (all P < 0.001); while si-WDFY3-AS2 transfection could decrease the protein levels of SOX2, OCT4 and Nanog (P < 0.01, P < 0.001, Fig. 3B). Furthermore, we also detected the CD44^+^, CD166^+^-positive population in A2780-DDP sphere-forming cells. As shown in Fig. 3C, WDFY3-AS2 overexpression significantly induced the number of CD44^+^, CD166^+^-positive cells in A2780-DDP tumorspheres (P < 0.001). These above data noted that WDFY3-AS2 seemed to be more efficient in the promotion of A2780-DDP cells tumorspheres.

### LncRNA WDFY3-AS2 sponged miR-139-5p in OC A2780-DDP cells

**Fig. 4:**
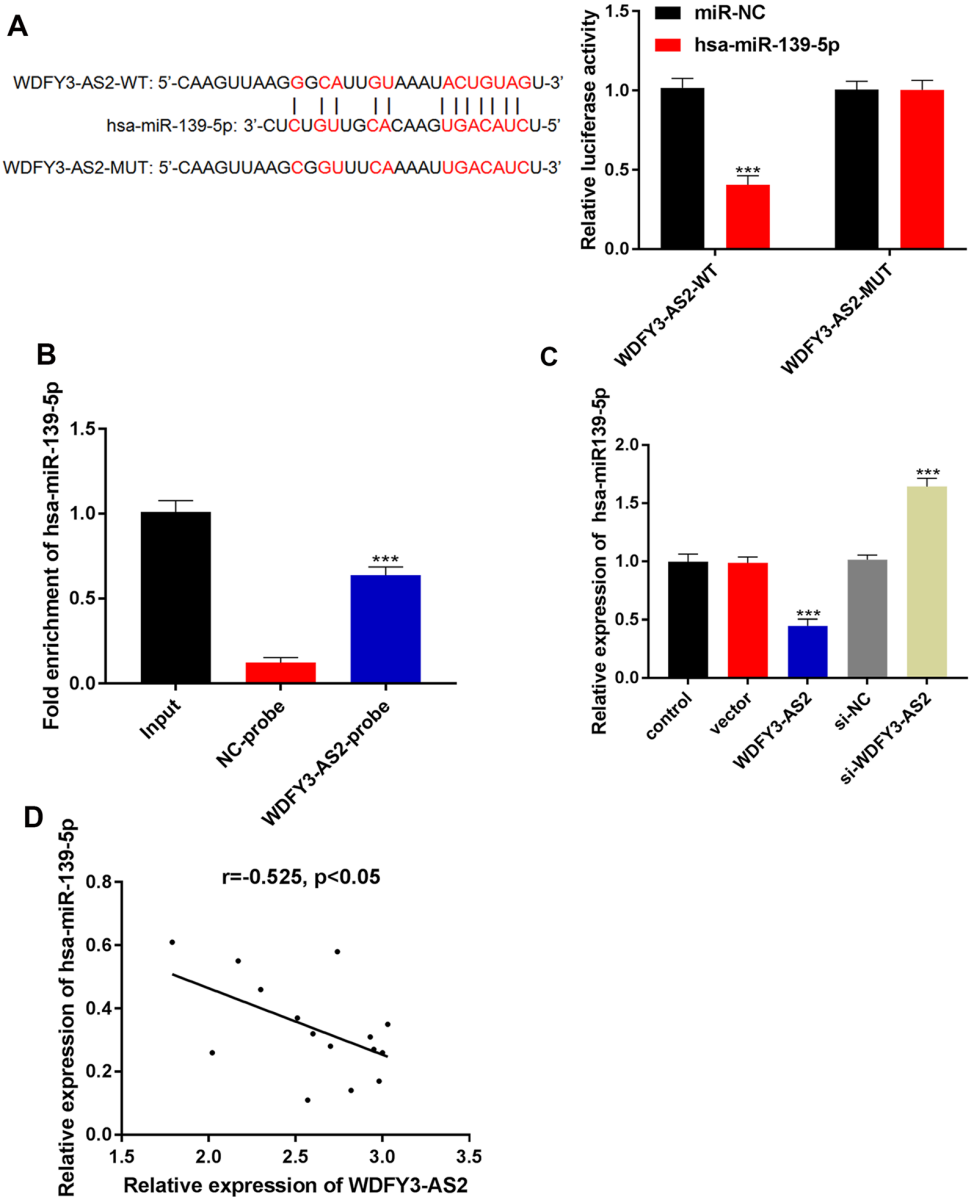
LncRNA WDFY3-AS2 sponged miR-139-5p in OC A2780-DDP cells. **A** Dual luciferase reporter assay validated the binding of miR-139-5p with lncRNA WDFY3-AS2. **B** RNA pull-down confirmed that the binding of miR-139-5p and lncRNA WDFY3-AS2 by the WDFY3-AS2-probe. **C** qRT-PCR analysis of the expression of miR-139-5p in A2780-DDP cells with WDFY3-AS2 or si-WDFY3-AS2 transfection. **D** Negative correlations between miR-139-5p and WDFY3-AS2. n = 15 per group, ***P < 0.001 vs. control

As lncRNAs can function by sequestering miRNAs and thus controlling gene expression, we next assessed possible miRNA binding partners for WDFY3-AS2. Starbase predicted WDFY3-AS2 to contain complementary sequences to the hsa-miR-139-5p seed region. In order to confirm these putative interactions, dual luciferase reporter assays were used and showed that WDFY3-AS2-WT significantly suppressed the activity of the reporter plasmid with miR-139-5p (P < 0.001, Fig. 4A). RNA pull down assay was used to detect the enrichment of miR-139-5p by the WDFY3-AS2 biotin probe (WDFY3-AS2-probe). The result confirmed that miR-139-5p enrichment by the WDFY3-AS2-probe was remarkably more than that by the NC-probe (Fig. 4B, P < 0.001). Besides, miR-139-5p expression was also reduced as measured via qRT-PCR in A2780-DDP cells transfected with WDFY3-AS2. MiR-139-5p expression was elevated in si-WDFY3-AS2 group than that in si-NC group (Fig. 4C, P < 0.001). In addition, correlation analyses demonstrated that levels of WDFY3-AS2 were negatively correlated with miR-139-5p in cisplatin-resistant OC tissues (r = − 0.525, P < 0.05 Fig. 4D). These data indicated that WDFY3-AS2 played a role in tumor progression via targeting miR-139-5p negatively.

### LncRNA WDFY3-AS2 upregulated SDC4 expression by sponging miR-139-5p in OC A2780-DDP cells

**Fig. 5:**
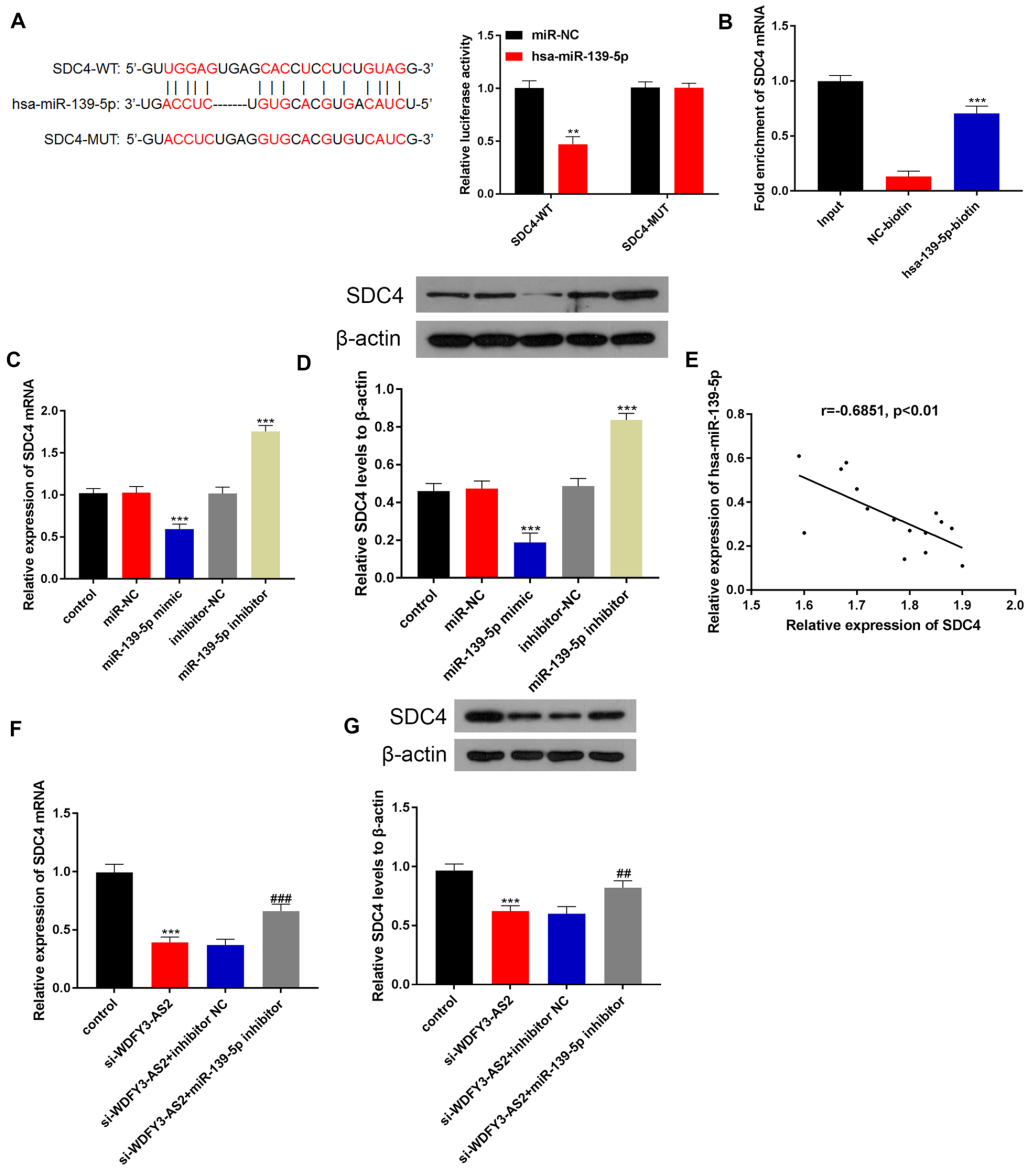
LncRNA WDFY3-AS2 upregulated SDC4 expression by sponging miR-139-5p in OC A2780-DDP cells. **A** Dual luciferase reporter assay validated miR-139-5p binding to SDC4. **B** RNA pull-down confirmed that the binding of miR-139-5p and SDC4 by the miR-139-5p-biotin. **C** qRT-PCR expression analysis of SDC4 in OC A2780-DDP cells. **D** Western blotting assessment of SDC4 protein expression in OC A2780-DDP cells. **E** The negative correlation between miR-139-5p and SDC4, n = 15. **F, G,** The mRNA and protein expression of SDC4 in si-WDFY3-AS2 transfected cells with co-transfection of miR-139-5p inhibitor. ^###^P < 0.001 vs. si-WDFY3-AS2 + inhibitor NC, ***P < 0.001 vs. control

We continued to find target gene that can bind with miR-139-5p by the Starbase. According to the Fig. 5A, we found potential binding site between miR-139-5p and SDC4 via dual-luciferase assay (P < 0.001). RNA pull down assay results suggested that SDC4 mRNA expression was significantly increased in miR-139-5p-biotin group compared to NC-biotin group (P < 0.001, Fig. 5B), indicating that miR-139-5p can directly bind to SDC4. We also examined how miR-139-5p affected SDC4 at the mRNA and protein levels via qRT-PCR and western blotting. SDC4 expression in A2780-DDP cells was inhibited by the miR-139-5p mimic, while SDC4 expression was suppressed in the miR-139-5p inhibitor group (Fig. 5C, D, P < 0.001). The correlation analysis demonstrated that SDC4 expression was negatively correlated with miR-139-5p (r = − 0.6851, P < 0.01 Fig. 5E, F). These findings indicated that SDC4 was targeted by miR-139-5p negatively. Furthermore, co-transfection of the miR-139-5p inhibitor could rescue the SDC4 expression reduced by si-WDFY3-AS2 in A2780-DDP cells (Fig. 5G, H, P < 0.001). Overall, these results indicated that WDFY3-AS2 functioned as ceRNA in regulating the expression of SDC4 by serving as a sponge for miR-139-5p in OC A2780-DDP cells.

### LncRNA WDFY3-AS2 promoted the cisplatin-resistant in OC A2780-DDP cells by controlling the miR-139-5p/SDC4 axis

**Fig. 6:**
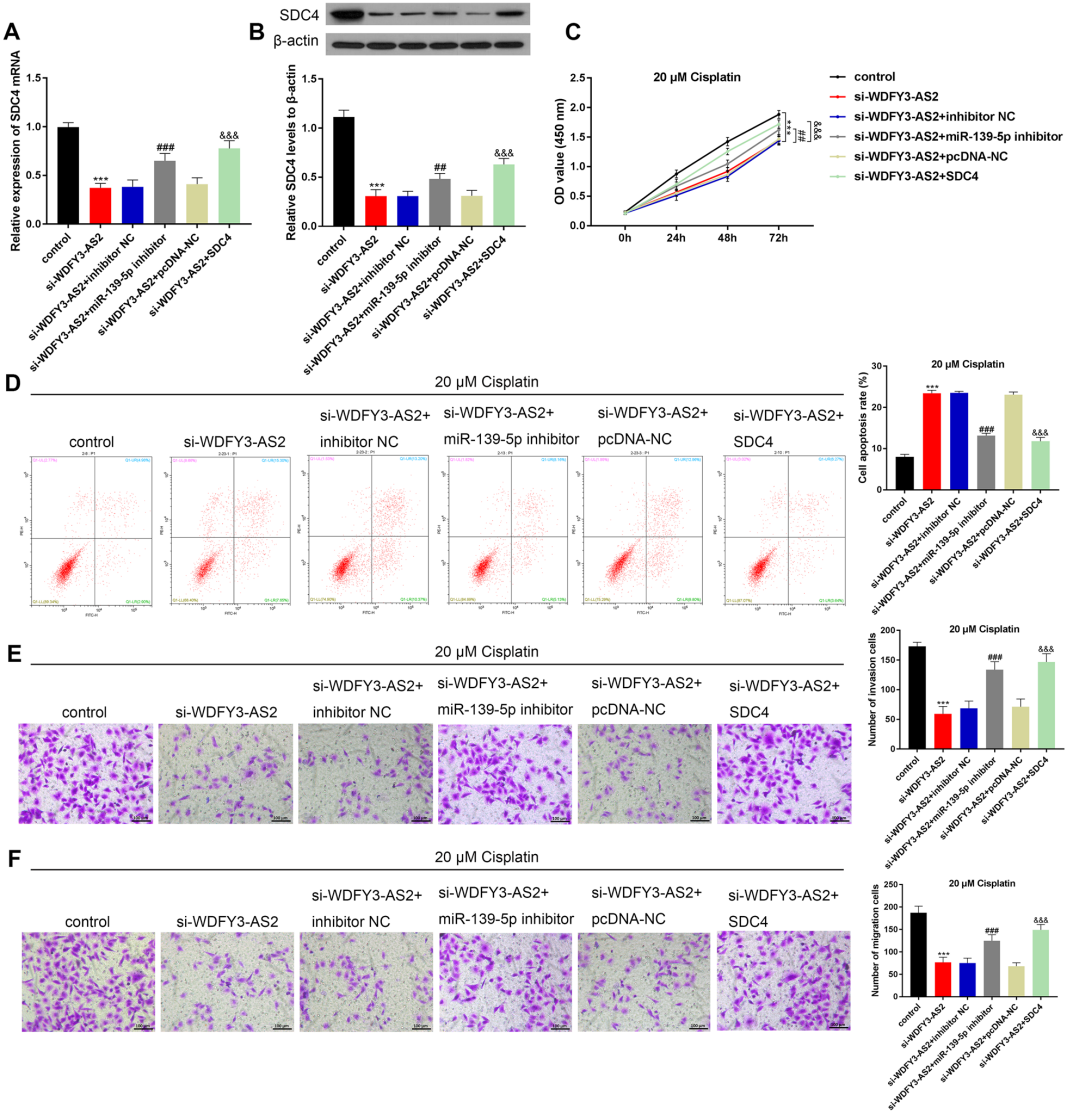
LncRNA WDFY3-AS2 promoted the cisplatin-resistant in OC A2780-DDP cells by up-regulating SDC4 by sponging miR-139-5p. **A**, **B** mRNA and protein levels of SDC4 in si-WDFY3-AS2 transfected cells with co-transfection of miR-139-5p inhibitor or pcDNA-SDC4 (SDC4) as measured via qRT-PCR and western blot. **C**, CCK8 results of the cell viability of transfected A2780-DDP cells. **D** OC A2780-DDP cells apoptosis after co-transfection and treatment of cisplatin as assessed via flow cytometry. **E**, **F** Transwell assay showed that the invasive and migration activity in A2780-DDP cells (100 μm). ***P < 0.001 vs. control, ^###^P < 0.001 vs. si-WDFY3-AS2 + inhibitor NC, ^&&&^P < 0.001 vs. si-WDFY3-AS2 + pcDNA-NC

To further investigate the WDFY3-AS2 regulation of cisplatin resistance in OC A2780-DDP cells via targeting miR-139-5p/SDC4, after the treatment of 20 μM cisplatin, rescue functional experiments were performed. Co-transfection of the miR-139-5p inhibitor or pcDNA-SDC4 (SDC4) could rescue the SDC4 expression reduced by si-WDFY3-AS2 in A2780-DDP cells (Fig. 6A, 6B, P < 0.001). CCK-8 was performed to measure viability. The cell growth ability was inhibited after si-WDFY3-AS2 transfection, but enhanced again after co-transfecting with miR-139-5p inhibitor or SDC4 up-regulation (Fig. 6C, P < 0.01, P < 0.001). Besides, miR-139-5p inhibitor or pcDNA-SDC4 (SDC4) contributed to a marked decrease the apoptotic rate induced by si-WDFY3-AS2 (Fig. 6D, P < 0.001). Additional, the transwell test manifested invasive and migrated cells reduced after si-WDFY3-AS2 transfection, but increased noticeably again after miR-139-5p inhibitor or SDC4 transfection (Fig. 6E, F, P < 0.001). Together, WDFY3-AS2 could mediate the cisplatin-resistant in OC A2780-DDP cells by up-regulating SDC4 by serving as a sponge for miR-139-5p.

### LncRNA WDFY3-AS2 induced the traits of CSCs by modulating the miR-139-5p/SDC4 axis

**Fig. 7:**
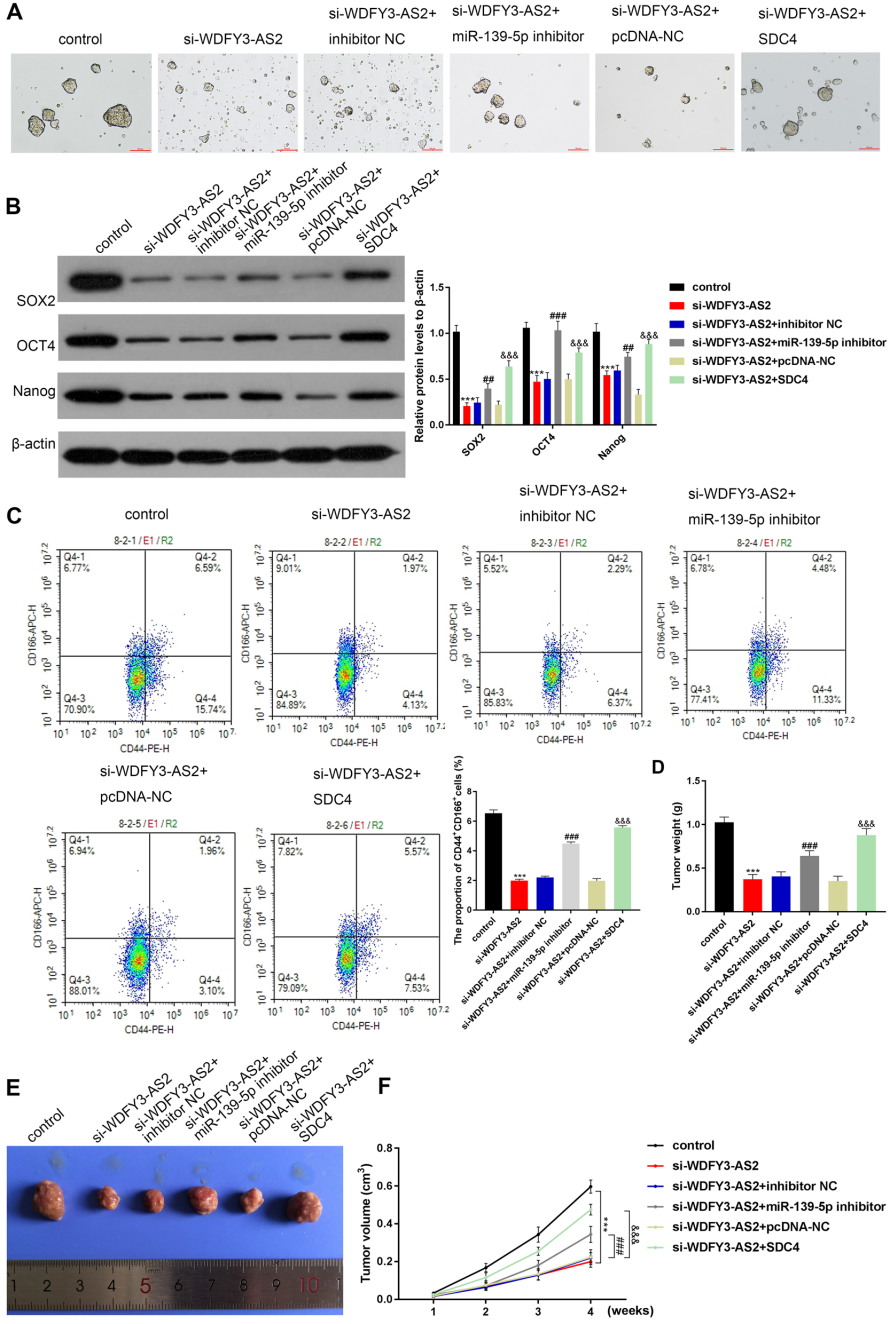
LncRNA WDFY3-AS2 induced the traits of CSCs by regulating miR-139-5p/SDC4. **A** Representative A2780-DDP sphere-forming cell images were acquired (100 μm). The transfected A2780-DDP cells were treated for 7 days with cisplatin (20 μM). **B** Western blotting was performed to assess CSC markers protein expression including the SOX2, OCT4 and Nanog in A2780-DDP sphere-forming cells. **C** CD44^+^, CD166^+^-positive cells in A2780-DDP sphere-forming cells detected via flow cytometry. **D** The weight of transplanted tumors in nude mice injected with si-WDFY3-AS2, miR-139-5p inhibitor or SDC4. **E** Assessment of tumors in nude mice. **F** The volume curve of xenograft tumors. ***P < 0.001 vs. control, ^###^P < 0.001 vs. si-WDFY3-AS2 + inhibitor NC, ^&&&^P < 0.001 vs. si-WDFY3-AS2 + pcDNA-NC

To explore the mechanism of WDFY3-AS2 on OC, after the treatment of 20 μM cisplatin, the si-WDFY3-AS2 transfected cells were co-transfected with the miR-139-5p inhibitor or pcDNA-SDC4 (SDC4). Seven days post treatment, co-transfection of the miR-139-5p inhibitor or pcDNA-SDC4 (SDC4) could rescue the inhibition of the formation of A2780-DDP tumorspheres by si-WDFY3-AS2 (Fig. 7A). We also detected significant decreases in lung CSC protein marker expression (SOX2, OCT4 and Nanog) after si-WDFY3-AS2 transfection, while, the miR-139-5p inhibitor or SDC4 could rescue those (P < 0.01, P < 0.001, Fig. 7B). Moreover, flow cytometry analysis was utilized to detect CD44^+^, CD166^+^-positive population in A2780-DDP sphere-forming cells. As illustrated in Fig. 7C, miR-139-5p inhibitor or SDC4 could rescue the number of CD44^+^, CD166^+^-positive cells decreased by si-WDFY3-AS2 in A2780-DDP tumorspheres (P < 0.001). These findings showed that WDFY3-AS2 induced the traits of CSCs by regulating miR-139-5p/SDC4. Furthermore, to assess whether the WDFY3-AS2 could regulate miR-139-5p to influence OC cell cisplatin resistance via SDC4 in vivo, cisplatin was intraperitoneally administered to nude mice harboring subcutaneous tumor xenografts. The weights and volumes of tumors in animals that had been administered the miR-139-5p inhibitor or SDC4 were notably increased relative to the si-WDFY3-AS2 mice (all P < 0.001, Fig. 7D–G).

## Discussion

OC is a gynecological tumor, and most advanced OC patients undergo combination surgical and platinum-based chemotherapeutic treatment using first-line agents such as cisplatin or carboplatin [32–35]. However, drug resistance often leads to therapy failure. In our study, we sought to explore the functions of lncRNA WDFY3-AS2 on cisplatin resistance in OC. We found that overexpression of the WDFY3-AS2 increased cell viability, migration, and invasion, inhibited apoptosis, as well as OC CSC traits induction, as shown by induced tumorsphere formation, CD133-positive cell numbers, and the expression levels of CSC markers both in vitro and in vivo. Moreover, we demonstrated that lncRNA WDFY3-AS2 sponged miR-139-5p to downregulate its expression, and WDFY3-AS2 increased SDC4 expression by sponging miR-139-5p. These findings suggested that WDFY3-AS2 influenced cisplatin resistance and OC-CSCs via regulation of miR-139-5p/SDC4 axis.

The dysregulation of lncRNAs is a common feature of OC wherein they can influence malignant cancer cell phenotypes [36]. WDFY3-AS2 expression level was reportedly low in both OC cells and tissues [26]. Also WDFY3-AS2 has been identified as a potential prognostic biomarker of diffuse glioma given that its overexpression in this cancer type is linked to longer patient OS [37]. The upregulation of WDFY3-AS2 has also been detected in lung adenocarcinoma (LUAD), with the knockdown of this lncRNA being sufficient to impair the proliferative, migratory, and invasive activity of LUAD cells while promoting their apoptotic death [28]. The role of WDFY3-AS2 in OC chemoresistance has not been assessed previously. Herein, we found that WDFY3-AS2 expression was increased in cisplatin resistant A2780-DDP OC cells compared to A2780 cells. si-WDFY3-AS2 inhibited the proliferative, migratory, invasive, and tumorsphere formation of OC A2780-DDP cells, while driving their apoptotic death. These results suggested that WDFY3-AS2 is a key regulator of the chemoresistance of OC. However, our observation was in contrast to prior findings that lncRNA WDFY3-AS2 suppresses tumor progression in ovarian cancer [26]. We speculate that this may be due to the specificity of ovarian tissue chemotherapy. The mechanisms of their associations require investigation in future studies.

In general, cytoplasmic lncRNAs can control gene expression via a ceRNA mechanism [38], and WDFY3-AS2 is expressed at high levels in the cytoplasm [26]. Through several experiments we found that WDFY3-AS2 sponged miR-139-5p and thereby downregulated it, while WDFY3-AS2 could upregulate SDC4 by sponging miR-139-5p. Many cancers exhibit the downregulation of miR-139-5p, which can in turn suppress the malignant properties of many cancer cells [39, 40]. Such miR-139-5p downregulation has been linked to multi-drug chemoresistance in breast, colorectal cancer [41, 42] and OC [43]. In this present study, we also found that significant miR-139-5p downregulation was evident in cisplatin-resistant OC A2780-DDP cells relative to their parental A2780 cells. The miR-139-5p inhibitor reversed the cisplatin resistance of OC induced by si-WDFY3-AS2 in vitro and in vivo. These data thus indicated that overexpressing WDFY3-AS2 regulated the cisplatin resistance in OC via targeting miR-139-5p.

CSCs are key mediators of oncogenesis in humans [44]. Although CSCs represent only about 2–5% population of cells in the tumor, they are reported to be chemoresistant and result in tumor relapse and recurrence [45, 46]. CSCs are characterized by their ability to for 3D spheres in serum-free media, and such spheroid-forming cell populations are considered to by CSC-enriched [47]. SDM tumorpshere formation can therefore be measured to readily assess the biology of CSCs [48]. CSCs are also distinguished by specific cell markers. SOX2, OCT4 and Nanog are considered pluripotent genes and stem cell markers to regulate CSC activity [49, 50]. CD44 and CD166 were reported as markers for adult stem cells [51, 52]. Herein, we employed a tumorsphere formation assay to enrich for A2780-DDP CSCs, revealing that these cells grown in SFM were able to form these spheroid structures and expressed higher levels of known CSC marker proteins (SOX2, OCT4, and Nanog) in addition to expressing higher levels of CD44/CD166. And we also found that the properties of CSCs in these cells were regulated by WDFY3-AS2 via miR-139-5p/SDC4 axis.

## Conclusions

In summary, these results indicated that WDFY3-AS2 may facilitate cisplatin resistance via the expression of miR-139-5p/SDC4 in the OC A2870-DDP cells in vitro and in vivo. Additionally, si-WDFY3-AS2 could lead to the decrease of the cell proliferation, migration, invasion and CSC-like state, and apoptosis induction in OC A2870-DDP cells. Our data thus highlight a novel target that may be modulated to overcome chemoresistance in OC.

## Supplementary Information

**Additional file 1:** The ceRNA network including the WDFY3-AS2 and miR-139-5p

## Data Availability

All data generated or analyzed during this study are included in this published article.

## Abbreviations

OC: Ovarian cancer
EOC: Epithelial ovarian cancer
CSCs: Cancer stem cells
SDC: Syndecan
