# Correction to: LncRNA WDFY3-AS2 promotes cisplatin resistance and the cancer stem cell in ovarian cancer by regulating hsa-miR-139-5p/SDC4 axis

**DOI:** 10.1186/s12935-023-03029-y

**Published:** 2023-08-23

**Authors:** Yue Wu, Ting Wang, Lin Xia, Mei Zhang

**Affiliations:** 1 Department of Integrated Chinese and Western Medicine Oncology, The First Affiliated Hospital of Anhui Medical University, No. 218, Jixi Road, Hefei, 230022 Anhui China; 2 The Traditional and Western Medicine (TCM)-Integrated Cancer Center of Anhui Medical University, 81 Meishan Road, Hefei, 230032 China; 3 Graduate School of Anhui, University of Traditional Chinese Medicine, Hefei, 230012 Anhui China

**Correction: Cancer Cell Int (2021) 21:284**

10.1186/s12935-021-01993-x

In the original article, the author would like to make the below changes in bold.

**Western blotting**.

RIPA buffer (Sigma, USA) was used **to isolate protein**, and there proteins were then separated by 10% SDS-PAGE, transferred to PVDF membranes (Millipore, MA, USA),

**Results**.

***The levels of lncRNA WDFY3AS2 in OC cells with different levels of cisplatin sensitivity***.

SDC4 expression in A2780-DDP cells was inhibited by the miR-139-5p mimic, while SDC4 expression was **increased** in the miR-139-5p inhibitor group (Fig. 5C, D, P < 0.001). The correlation analysis demonstrated that SDC4 expression was negatively correlated with miR-139-5p (r=-0.6851, P < 0.01 **Fig. 5E**). These findings indicated that SDC4 was targeted by miR-139-5p negatively. Furthermore, co-transfection of the miR-139-5p inhibitor could rescue the SDC4 expression reduced by si-WDFY3-AS2 in A2780-DDP cells (Fig. **5F,G**, P < 0.001).

The weights and volumes of tumors in animals that had been administered the miR-139-5p inhibitor or SDC4 were notably increased relative to the si-WDFY3-AS2 mice (all P < 0.001, **Fig. 7D-F**).

We found that overexpression of the WDFY3-AS2 increased cell viability, migration, and invasion, inhibited apoptosis, as well as OC CSC traits induction, as shown by induced tumor sphere formation, **CD44-,CD166-**positive cell numbers, and the expression levels of CSC markers both in vitro and in vivo.

