# Supplementary figures and images for: LncRNA WDFY3-AS2 promotes cisplatin resistance and the cancer stem cell in ovarian cancer by regulating hsa-miR-139-5p/SDC4 axis
Source: Cancer Cell Int. 2021 May 29;21:284. doi: 10.1186/s12935-021-01993-x (PMC8164817; doi:10.1186/s12935-021-01993-x)

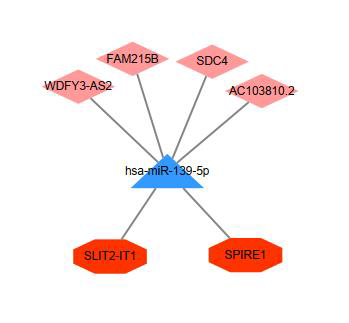

Supplement: Supplementary file 1 — Additional file 1: The ceRNA network including the WDFY3-AS2 and miR-139-5p [file 12935_2021_1993_MOESM1_ESM.tif]
